# Applications of Electrochromic Copolymers Based on Tris(4-carbazoyl-9-ylphenyl)amine and Bithiophene Derivatives in Electrochromic Devices

**DOI:** 10.3390/ma11101895

**Published:** 2018-10-03

**Authors:** Chung-Wen Kuo, Jui-Cheng Chang, Po-Ying Lee, Tzi-Yi Wu, Yu-Chang Huang

**Affiliations:** 1 Department of Chemical and Materials Engineering, National Kaohsiung University of Science and Technology, Kaohsiung 80778, Taiwan; welly@nkust.edu.tw (C.-W.K.); k40017105@gcloud.csu.edu.tw (P.-Y.L.); ych@nkust.edu.tw (Y.-C.H.); 2 Bachelor Program in Interdisciplinary Studies, National Yunlin University of Science and Technology, Yunlin 64002, Taiwan; d700215@gmail.com; 3 Department of Chemical Engineering and Materials Engineering, National Yunlin University of Science and Technology, Yunlin 64002, Taiwan

**Keywords:** copolymer, electrochemical polymerization, spectroelectrochemistry, electrochromic device, redox stability

## Abstract

Four copolymers (P(tCz (tris(4-carbazoyl-9-ylphenyl)amine)-*co*-bTP (2,2′-bithiophene)), P(tCz-*co*-CPDT (4H-cyclopenta[2,1-b:3,4-b’]dithiophene)), P(tCz-*co*-DTC (3,6-di(2-thienyl)carbazole)), and P(tCz-*co*-CPDTK (cyclopentadithiophene ketone))) are deposited on indium tin oxide (ITO) surfaces using electrochemical polymerization. Spectroelectrochemical properties of copolymer electrodes reveal that the colors of P(tCz-*co*-bTP) film are pinkish-orange, light olive green, light grayish blue, and dark blue at 0.0, 0.8, 1.2, and 1.6 V, respectively, whereas the color variations of P(tCz-*co*-CPDTK) film are light yellow, yellow, and blue at 0.0 V, 0.8 V, and 1.5 V, respectively. The Δ*T* of P(tCz-*co*-bTP), P(tCz-*co*-CPDT), P(tCz-*co*-DTC), and P(tCz-*co*-CPDTK) films are estimated to be 43.0% at 967 nm, 28.7% at 864 nm, 43.6% at 870 nm, and 24.5% at 984 nm, respectively. Five electrochromic devices (ECDs) are assembled using the tCz-based homopolymer and copolymers as coloring electrodes, and poly(2,2-dimethyl-3,4-propylenedioxythiophene) (PProDOT-Me_2_) as the complementary electrode. P(tCz-*co*-DTC)/PProDOT-Me_2_ ECD reveals high transmittance change (45.9% at 624 nm), P(tCz-*co*-CPDTK)/PProDOT-Me_2_ ECD shows high *η* (513.0 cm^2^ C^−1^ at 582 nm), and P(tCz-*co*-bTP)/PProDOT-Me_2_ ECD presents short switching time (less than 0.4 s) at 628 nm. Moreover, these ECDs show satisfactory redox stability and open circuit stability.

## 1. Introduction 

Electrochromic materials (ECMs) have attracted attention in the scientific communities due to their advantage of low driving voltage and high contrast of transmittance [1]. In around 1960–1970, ECMs have been widely investigated for potential use in automotive rearview mirrors, smart windows, and sunroofs. The most studied electrochromic materials are metal oxides, metal phthalocyanines, metal coordination complexes, viologens, and conjugated polymers [2]. Among these ECMs, conjugated polymers show high coloration efficiency and short response time. The electrochromic behaviors of conjugated polymers can be tuned by proper incorporations of specific substituents in polymer backbones [3].

The commonly used conjugated polymers of ECMs contain polythiophene [4,5], polycarbazole [6,7], polytriphenylamine [8,9], polyaniline [10], polyindole [11,12], and poly(3,4-ethylenedioxythiophene) (PEDOT) [13]. Polycarbazoles have been extensively studied for various optical and electrochemical devices due to their superior electroactive properties. Polycarbazoles can be functionalized at the 3,6-, 2,7-, and 9-positions of carbazole groups [14,15]. Polythiophene can be easily modified to offer a wide variety of useful optical and electrochemical properties such as tunable band gap, conductivity, and oxidation and reduction activity [16,17]. Jia et al. [18] reported the electrochromic properties of poly(carbazoyltriphenylamine) (poly(CBZ-TPA)) and poly(carbazoyltriphenylaminethiophene) (poly(CBZ-TPA-Th)) films. Poly(CBZ-TPA) film displayed camel gray, light gray, and army green at −0.5, 0, and 1.6 V, respectively, whereas poly(CBZ-TPA-Th) film exhibited maize-yellow, milk white, and dark green at −0.5, 0, and 1.4 V, respectively. Yigit et al. [19] reported the spectroelectrochemical characterization of azobenzene- and coumarin-containing polymers (poly[9-(2-4-(phenyldiazenyl) phenoxy) ethyl-3,6-di(thiophen-2-yl)-9H-car-bazole] (PTCbzAz), poly[3,6-bis(2,3-dihydrothieno[3,4-b][1,4]di-oxin-5-yl)-9-(2-(4-(phenyldiazenyl)phenoxy) ethyl)-9H-carbazole] (PECbzAz), poly[4-(2-(3,6-di(thiophen-2-yl)-9H-carba-zol-9-yl) ethoxy)-2H-chromen-2-one] (PTCbzCo) and poly[3,6-bis (2,3-dihydrothieno[3,4-b][1,4]dioxin-5-yl)-9H-(carbazol-9-yl)ethoxy-2H-chromen-2-one] (PECbzCo)). The spectroelectrochemical studies showed that the incorporations of azobenzene and coumarin units in polymer backbone gave rise to multicolored electrochromisms. Moreover, PProDOT-Me_2_ is one of the promising polythiophene derivatives. PProDOT-Me_2_ has been employed as the cathodically coloring material of ECDs due to PProDOT-Me_2_ film being transparent in the oxidized state and the resulting deep color in the reduced state [20]. Furthermore, copolymerization is a promising way to tune the electrochromic characteristics of polymer films. Copolymerization of various monomers containing specific functional groups can bring about interesting electrochromic behaviors. For this matter, four carbazole- and bithiophene-containing copolymers (P(tCz-*co*-bTP (2,2′-bithiophene)), P(tCz-*co*-CPDT), P(tCz-*co*-DTC (3,6-di(2-thienyl)carbazole)), and P(tCz-*co*-CPDTK (cyclopentadithiophene ketone))) are copolymerized electrochemically to study their promising applications as ECMs. tCz contains three carbazole units linked by a triphenylamine core. The presence of three oxidizable carbazole and one oxidizable triphenylamine groups in tCz unit facilitates the formations of polaron and bipolaron in the oxidized states of tCz-based polymer films. All of bTP, CPDT, DTC, and CPDTK units comprise two thiophene rings linked by specific groups. The two thiophene rings of bTP and DTC units are linked by a single bond and a carbazole group, respectively. CPDT comprises two thiophene rings linked by a single bond and a methylene bridge at the 2- and 3-positions of thiophene rings. CPDTK contains two thiophene rings linked by a carbonyl group and a single bond at the 2- and 3-positions of thiophene rings. The electron-withdrawing carbonyl group in CPDTK unit can diminish the LUMO level and band gap of polymers, which is useful in electrochromic applications. Moreover, five ECDs comprised of PtCz, P(tCz-*co*-bTP), P(tCz-*co*-CPDT), P(tCz-*co*-DTC), or P(tCz-*co*-CPDTK) as anodically coloring layer, and PProDOT-Me_2_ as cathodically coloring layer were constructed and their spectroelectrochemical behaviors, transmittance variations of electrochromic switching, optical memory, and redox stability were also investigated.

## 2. Experimental

### 2.1. Materials

2,2′-bithiophene (bTP), CPDT, and CPDTK were purchased from Luminescence Technology Corp. (Taipei, Taiwan). ProDOT-Me_2_, DTC, and tCz were synthesized according to previously published procedures [21,22,23].

### 2.2. Electrosynthesis of PtCz, P(tCz-co-bTP), P(tCz-co-CPDT), P(tCz-co-DTC), P(tCz-co-CPDTK), and PProDOT-Me_2_ Films

The electrosynthesis of PtCz, P(tCz-*co*-bTP), P(tCz-*co*-CPDT), P(tCz-*co*-DTC), and P(tCz-*co*-CPDTK) films was carried out in a 0.2 M LiClO_4_/acetonitrile (ACN)/dichloromethane (DCM) solution, and the feed molar ratio of monomers are presented in Table 1. The PtCz, P(tCz-*co*-bTP), P(tCz-*co*-CPDT), P(tCz-*co*-DTC), and P(tCz-*co*-CPDTK) films were prepared potentiodynamically by scanning the potential between 0.0 V and 1.8 V (vs. Ag/AgCl in 3 M KCl solution) for 3 cycles. The PProDOT-Me_2_ film was prepared using 2 mM ProDOT-Me_2_ in a 0.2 M LiClO_4_-containing ACN solution. PProDOT-Me_2_ film was deposited potentiostatically at 1.7 V (vs. Ag/AgCl in 3 M KCl solution).

### 2.3. Electrochemical and Spectroelectrochemical Characterizations

Electrochemical characterizations of PtCz, P(tCz-*co*-bTP), P(tCz-*co*-CPDT), P(tCz-*co*-DTC), P(tCz-*co*-CPDTK) films and their corresponding ECDs were performed using a CHI627D electrochemical analyzer (CH Instruments, Austin, TX, USA). The active areas of PtCz, P(tCz-*co*-bTP), P(tCz-*co*-CPDT), P(tCz-*co*-DTC), P(tCz-*co*-CPDTK), and PProDOT-Me_2_ films on ITO coated glass plate were 1.5 cm^2^. The characterizations of polymer films in ACN/DCM solution were carried out using Ag/AgCl and platinum wire as the reference and counter electrodes, respectively. Spectroelectrochemical characterizations were performed using an Agilent Cary 60 UV (Ultraviolet)-Visible spectrophotometer (Varian Inc., Walnut Creek, CA, USA) and a CHI627D electrochemical analyzer.

### 2.4. Construction of Electrochromic Devices

The electrolyte of ECD was prepared using poly(methyl methacrylate) (PMMA), propylene carbonate (PC), and LiClO_4_, the weight ratio of PMMA:PC:LiClO_4_ is 33:53:14. Electrochromic devices were fabricated using PtCz, P(tCz-*co*-bTP), P(tCz-*co*-CPDT), P(tCz-*co*-DTC), or P(tCz-*co*-CPDTK) as the anodic coloring layer and PProDOT-Me_2_ as the cathodic coloring layer. Both anodic coloring and cathodic coloring materials were electrodeposited on ITO glasses, respectively. The ECDs were constructed by arranging the anodic and cathodic coloring layers to face each other, and they were separated by the PMMA/PC/LiClO_4_ electrolyte.

## 3. Results and Discussion

### 3.1. Electrochemical Polymerization

**Figure 1:**
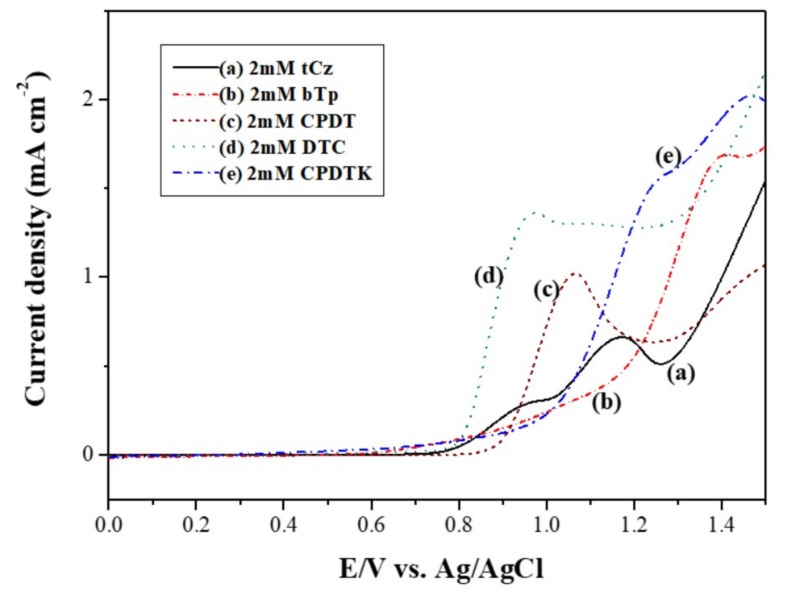
Electrooxidation of (**a**) 2 mM tCz; (**b**) 2 mM bTP; (**c**) 2 mM CPDT; (**d**) 2 mM DTC; and (**e**) 2 mM CPDTK at a scan rate of 100 mV s^−1^.

Figure 1 showed the electrooxidation of 2 mM tCz, 2 mM bTP, 2 mM CPDT, 2 mM DTC, and 2 mM CPDTK in 0.2 M LiClO_4_/ACN/DCM solution. The onset potential of oxidation for tCz, bTP, CPDT, DTC, and CPDTK were 0.78, 0.83, 0.89, 0.79, and 0.93 V, respectively. CPDTK showed higher onset potential of oxidation than those of bTP and CPDT, indicating the incorporation of electron withdrawing ketone group in CPDTK increasing the onset potential of oxidation significantly. The discrepancies between tCz vs. bTP, tCz vs. CPDT, tCz vs. DTC, and tCz vs. CPDTK were less than 0.15 V, implying that the copolymerizations of tCz vs. bTP, tCz vs. CPDT, tCz vs. DTC, and tCz vs. CPDTK are workable [24].

**Figure 2:**
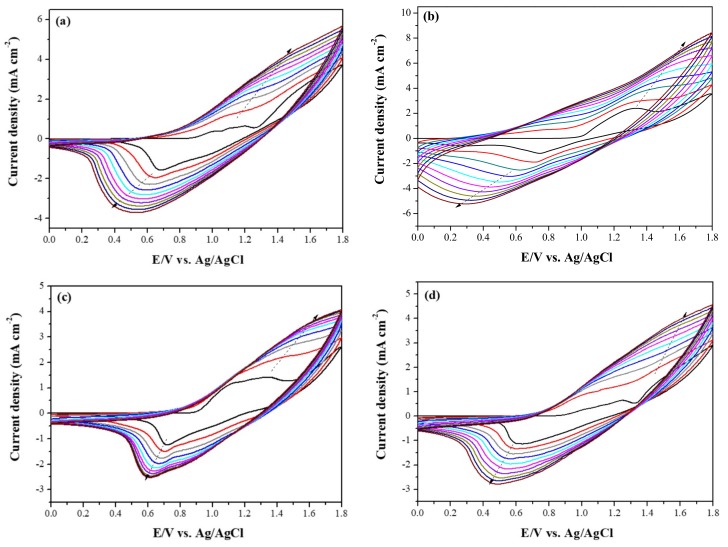
Electrochemical synthesis of (**a**) P(tCz-*co*-bTP); (**b**) P(tCz-*co*-CPDT); (**c**) P(tCz-*co*-DTC); and (**d**) P(tCz-*co*-CPDTK) in 0.2 M LiClO_4_/ACN/DCM solution.

**Figure 3:**
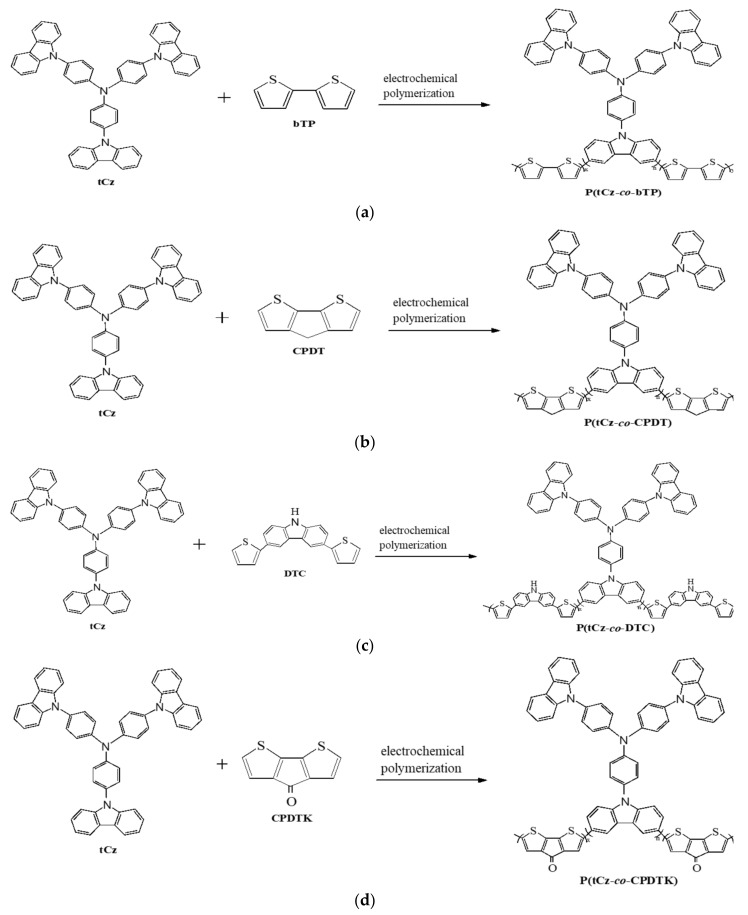
The polymerization schemes of (**a**) P(tCz-*co*-bTP); (**b**) P(tCz-*co*-CPDT); (**c**) P(tCz-*co*-DTC); and (**d**) P(tCz-*co*-CPDTK).

Figure 2 shows the electrochemical synthesis of copolymer films in a solution. The redox peak current densities increased with the increasing number of cycles, indicating the formation of polymer films on ITO substrates [25]. The oxidation peaks of P(tCz-*co*-bTP), P(tCz-*co*-CPDT), P(tCz-*co*-DTC), and P(tCz-*co*-CPDTK) located at around 1.10, 1.35, 1.45, and 1.10 V, respectively, and the reduction peaks of P(tCz-*co*-bTP), P(tCz-*co*-CPDT), P(tCz-*co*-DTC), and P(tCz-*co*-CPDTK) located at around 0.65, 0.73, 0.70, and 0.60 V, respectively. The polymerization schemes of P(tCz-*co*-bTP), P(tCz-*co*-CPDT), P(tCz-*co*-DTC), and P(tCz-*co*-CPDTK) are displayed in Figure 3.

### 3.2. Electrochemical Behavior of P(tCz-co-DTC) Film

**Figure 4:**
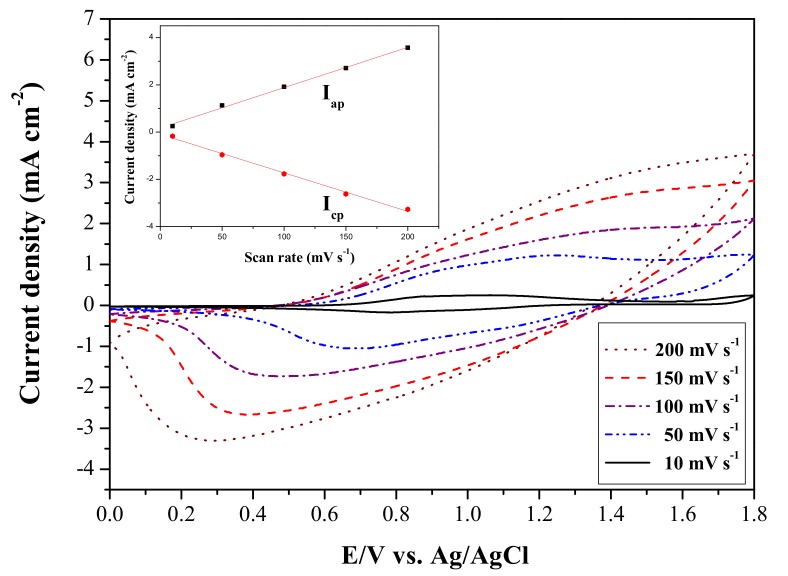
CV curves of the P(tCz-*co*-DTC) film at different scan rates. Inset: Scan rate dependence of the peak current densities of P(tCz-*co*-DTC) film.

The P(tCz-*co*-DTC) film was synthesized using the electropolymerization of tCz and DTC monomers were scanned at different scan rate in 0.2 M LiClO_4_/ACN/DCM solution. As shown in Figure 4, cyclic voltammogram (CV) curves of P(tCz-*co*-DTC) film showed well-defined redox peaks and the peak current densities increased linearly with the increasing scan rates (inset in Figure 4), demonstrating that the redox process is electroactively non-diffusional limited and P(tCz-*co*-DTC) film has adhered well to the ITO electrode [26].

### 3.3. Spectroelectrochemical Studies of Polymer Films

**Figure 5:**
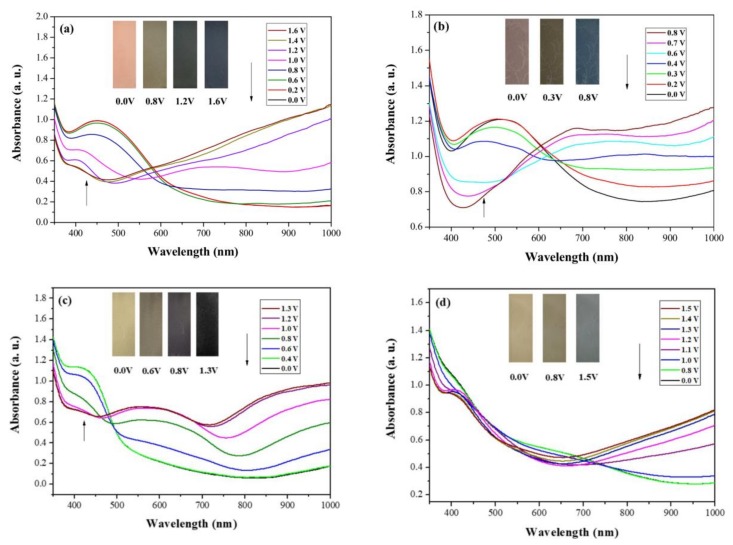
UV-Visible spectra of (**a**) P(tCz-*co*-bTP); (**b**) P(tCz-*co*-CPDT); (**c**) P(tCz-*co*-DTC); and (**d**) P(tCz-*co*-CPDTK) electrodes in 0.2 M LiClO_4_/ACN/DCM solution.

P(tCz-*co*-bTP), P(tCz-*co*-CPDT), P(tCz-*co*-DTC), and P(tCz-*co*-CPDTK) films were coated on ITO substrate and the spectral variations at various voltages were monitored using a UV-Vis spectrophotometer. As shown in Figure 5a, P(tCz-*co*-bTP) film has an absorption peak at 450 nm in the neutral state at 0.0 V, which can be referred to the π-π* transition of P(tCz-*co*-bTP), and P(tCz-*co*-bTP) film being light orange in the neutral state.

Upon increasing the voltage in the positive direction, the new absorption band at 967 nm emerged gradually, which are assigned to the formation of polaron and bipolaron absorption bands [27]. The colors of P(tCz-co-bTP) film were dark yellow, light blue and dark blue at 0.8, 1.2, and 1.6 V, respectively. In similar condition, P(tCz-co-CPDT), P(tCz-co-DTC), and P(tCz-co-CPDTK) films showed the maximum absorption at 500, 420, and 410 nm in the neutral state, respectively. This may be attributed to the effective conjugation of the polymer chains [28]. The colors of P(tCz-co-CPDT) film were light purple at 0.0 V, green at 0.3 V, and dark blue at 0.8 V, whereas the color variations of P(tCz-co-DTC) film were light yellow at 0.0 V, yellow at 0.6 V, blue at 0.8 V, and black at 1.3 V, the color variations of P(tCz-co-CPDTK) film were light yellow at 0.0 V, yellow at 0.8 V, and blue at 1.5 V. Table 2 shows the colorimetric values of P(tCz-co-bTP), P(tCz-co-CPDT), P(tCz-co-DTC), and P(tCz-co-CPDTK) at various applied potentials.

### 3.4. Electrochemical Switching of Polymer Films

**Figure 6:**
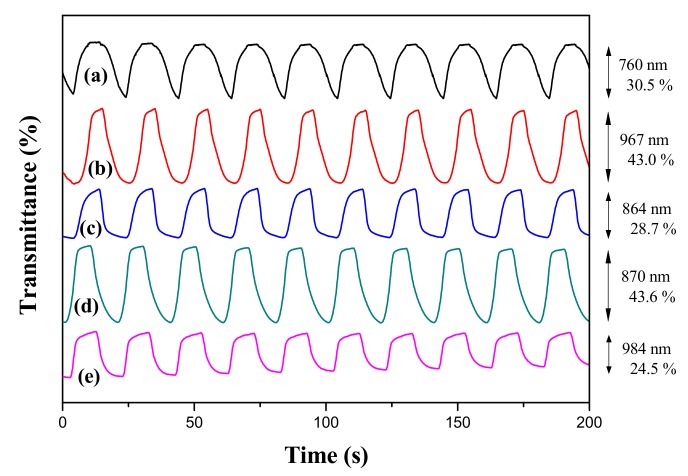
Transmittance-time profiles of (**a**) PtCz; (**b**) P(tCz-*co*-bTP); (**c**) P(tCz-*co*-CPDT); (**d**) P(tCz-*co*-DTC); and (**e**) P(tCz-*co*-CPDTK) electrodes in a solution.

Figure 6 shows the electrochromic switching properties of PtCz, P(tCz-*co*-bTP), P(tCz-*co*-CPDT), P(tCz-*co*-DTC), and P(tCz-*co*-CPDTK) films in a 0.2 M LiClO_4_/ACN/DCM solution, and the optical contrast (Δ*T*%) and ΔOD of polymer films were listed in Table 3. The polymer films were switched by potentials between 0.0 and 1.5 V with a time interval of 10 s. The Δ*T* of PtCz, P(tCz-*co*-bTP), P(tCz-*co*-CPDT), P(tCz-*co*-DTC), and P(tCz-*co*-CPDTK) films were estimated to be 30.5% at 760 nm, 43.0% at 967 nm, 28.7% at 864 nm, 43.6% at 870 nm, and 24.5% at 984 nm, respectively. P(tCz-*co*-bTP) and P(tCz-*co*-DTC) films showed a higher Δ*T* than those of PtCz, P(tCz-*co*-CPDT), and P(tCz-*co*-CPDTK) films, implying the incorporation of bTP and DTC units in the copolymer backbone enhances Δ*T* significantly. The response time required to reach 90% of entire transmittance change were determined to be 3.5–6.5 s for these polymer films. 

The *η* can be determined using the formula [29]:

(1) $$\eta =\frac{\Delta OD}{Q_{d}}$$

where *Q*_d_ refers to the amount of injected/ejected charge per unit active area. The *η* values of PtCz, P(tCz-*co*-bTP), P(tCz-*co*-CPDT), P(tCz-*co*-DTC), and P(tCz-*co*-CPDTK) films are calculated to be 42.6 cm^2^ C^−1^ at 760 nm, 67.6 cm^2^ C^−1^ at 967 nm, 76.7 cm^2^ C^−1^ at 864 nm, 78.3 cm^2^ C^−1^ at 870 nm, and 62.5 cm^2^ C^−1^ at 984 nm, respectively. Copolymer films show higher *η* values than that of PtCz film in a solution.

As shown in Table 4, P(tCz-*co*-DTC) film showed higher transmittance change than those reported for poly(ethyl-4-(3,6-di(thiophen-2-yl)-9*H*-carbazole-9-yl)-benzoate) (PETCB) film at 1100 nm [15], poly(9*H*-carbazol-9-ylpyrene) (PMCzP) film at 460 nm [30], and poly(1,3-bis(carbazol-9-yl)benzene) (PBCz) film at 1050 nm [31]. However, P(tCz-*co*-DTC) film showed a lower transmittance change than those reported for P(NO_2_-3Cz) film at 710 nm [14]. On the other hand, P(tCz-*co*-DTC) film revealed higher *η* than that reported for P(NO_2_-3Cz) [14], whereas P(tCz-*co*-DTC) film presented lower *η* than that reported for PBCz [31].

### 3.5. Spectroelectrochemistry of ECDs

**Figure 7:**
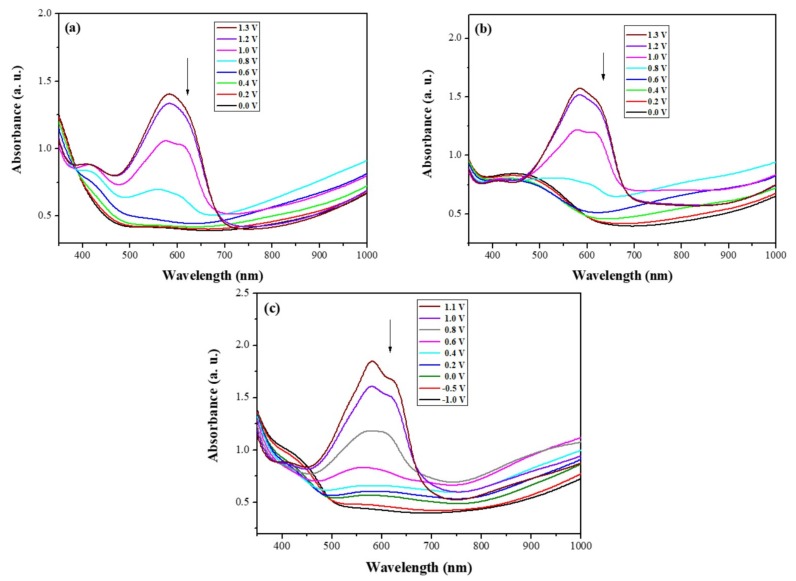
UV-Visible spectra of (**a**) PtCz/PProDOT-Me_2_; (**b**) P(tCz-*co*-bTP)/PProDOT-Me_2_; and (**c**) P(tCz-*co*-DTC)/PProDOT-Me_2_ ECDs.

Figure 7 shows the UV-Vis spectra of dual-type PtCz/PProDOT-Me_2_, P(tCz-*co*-bTP)/PProDOT-Me_2_, and P(tCz-*co*-DTC)/PProDOT-Me_2_ ECDs at various applied potentials. At 0.0 V, PtCz, P(tCz-*co*-bTP), and P(tCz-*co*-DTC) films showed a bleached color in their neutral state and PProDOT-Me_2_ was transparent in its oxidized state. 

Accordingly, PtCz/PProDOT-Me_2_, P(tCz-*co*-bTP)/PProDOT-Me_2_, and P(tCz-*co*-DTC)/PProDOT-Me_2_ ECDs revealed a bleached color at 0.0 V. Upon increasing the applied voltage from 0 to 1.3 V gradually, the absorption bands at 583–628 nm were emerged due to the absorption of PtCz, P(tCz-*co*-bTP), and P(tCz-*co*-DTC) films in their oxidized state and PProDOT-Me_2_ film in its neutral state. The colors of PtCz/PProDOT-Me_2_, P(tCz-*co*-bTP)/PProDOT-Me_2_, and P(tCz-*co*-DTC)/PProDOT-Me_2_ ECDs were dark blue at 1.3 V. Table 5 showed the photographs and colorimetric values of P(tCz-*co*-bTP)/PProDOT-Me_2_ and P(tCz-*co*-DTC)/PProDOT-Me_2_ ECDs at various potentials. P(tCz-*co*-bTP)/PProDOT-Me_2_ ECD revealed light tan, light brown, purple, blue, and dark blue at 0.0, 0.4, 0.6, 0.8, and 1.3 V, respectively, whereas P(tCz-*co*-DTC)/PProDOT-Me_2_ ECD displayed light beige, grey, light grey, blue, and dark blue at −1.0, 0.0, 0.4, 0.6, and 1.1 V, respectively.

### 3.6. Electrochemical Switching of ECDs

**Figure 8:**
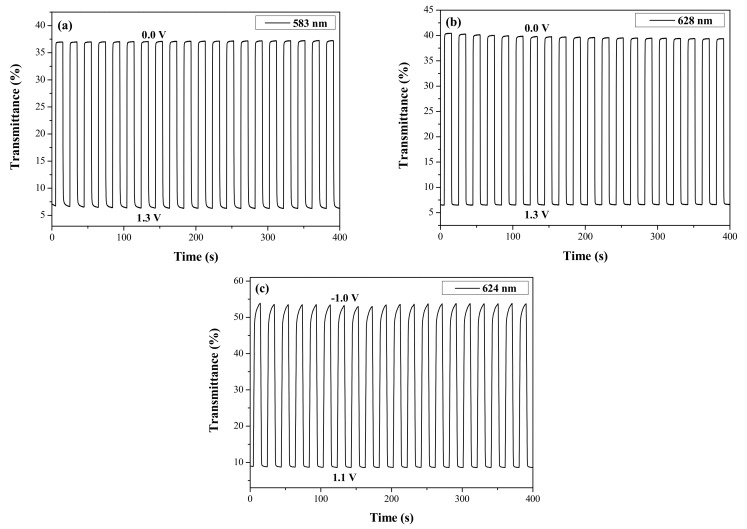
Transmittance-time profiles of (**a**) PtCz/PProDOT-Me_2_; (**b**) P(tCz-*co*-bTP)/PProDOT-Me_2_; and (**c**) P(tCz-*co*-DTC)/PProDOT-Me_2_ ECDs carried out by stepping potentials between bleached and colored states with a residence time of 10 s.

The double-potential-step chronoamperometry experiments of PtCz/PProDOT-Me_2_, P(tCz-*co*-bTP)/PProDOT-Me_2_, and P(tCz-*co*-DTC)/PProDOT-Me_2_ ECDs were implemented by stepping potentials between bleached and colored states, the time interval being 10 s. Figure 8 showed the transmittance-time profiles of PtCz/PProDOT-Me_2_, P(tCz-*co*-bTP)/PProDOT-Me_2_, and P(tCz-*co*-DTC)/PProDOT-Me_2_ ECDs, and the Δ*T*, *η*, and the switching time of PtCz/PProDOT-Me_2_, P(tCz-*co*-bTP)/PProDOT-Me_2_, P(tCz-*co*-CPDT)/PProDOT-Me_2_, P(tCz-*co*-DTC)/PProDOT-Me_2_ and P(tCz-*co*-CPDTK)/PProDOT-Me_2_ ECDs were presented in Table 6. The Δ*T* of PtCz/PProDOT-Me_2_, P(tCz-*co*-bTP)/PProDOT-Me_2_, P(tCz-*co*-CPDT)/PProDOT-Me_2_, P(tCz-*co*-DTC)/PProDOT-Me_2_ and P(tCz-*co*-CPDTK)/PProDOT-Me_2_ ECDs were estimated to be 30.9% at 583 nm, 32.0% at 628 nm, 19.7% at 582 nm, 45.9% at 624 nm, and 29.5% at 582 nm, respectively. P(tCz-*co*-DTC)/PProDOT-Me_2_ ECD showed the highest Δ*T*, and P(tCz-*co*-bTP)/PProDOT-Me_2_ ECD showed higher Δ*T* than that of PtCz/PProDOT-Me_2_ ECD, indicating that the incorporation of copolymers (P(tCz-*co*-bTP) and P(tCz-*co*-DTC)) as the anodically coloring layers leading to a higher Δ*T* than that of the homopolymer (PtCz). In similar situation, P(tCz-*co*-bTP)/PProDOT-Me_2_ and P(tCz-*co*-DTC)/PProDOT-Me_2_ ECDs showed higher ΔOD than that of PtCz/PProDOT-Me_2_ ECD. On the other side, the *η* of dual-type PtCz/PProDOT-Me_2_, P(tCz-*co*-bTP)/PProDOT-Me_2_, P(tCz-*co*-CPDT)/PProDOT-Me_2_, P(tCz-*co*-DTC)/PProDOT-Me_2_ and P(tCz-*co*-CPDTK)/PProDOT-Me_2_ ECDs were 437.4 cm^2^ C^−1^ at 583 nm, 387.0 cm^2^ C^−1^ at 628 nm, 419.7 cm^2^ C^−1^ at 582 nm, 400.5 cm^2^ C^−1^ at 624 nm, and 513.0 cm^2^ C^−1^ at 582 nm, respectively. P(tCz-*co*-CPDTK)/PProDOT-Me_2_ ECD showed the highest *η* among these ECDs. The *τ*_c_ and *τ*_b_ estimated for dual-type PtCz/PProDOT-Me_2_, P(tCz-*co*-bTP)/PProDOT-Me_2_, P(tCz-*co*-CPDT)/PProDOT-Me_2_, P(tCz-*co*-DTC)/PProDOT-Me_2_, and P(tCz-*co*-CPDTK)/PProDOT-Me_2_ ECDs are also shown in Table 6. The *τ*_s_ of dual-type ECDs were shorter than those of their corresponding anodic polymer films, indicating that the dual-type ECDs revealed a short distance between the two electrodes. P(tCz-*co*-bTP)/PProDOT-Me_2_ ECD showed the shortest *τ* among these ECDs.

Table 7 shows the comparisons of Δ*T*_max_ and *η*_max_ with reported ECDs. P(tCz-*co*-DTC)/PProDOT-Me_2_ ECD shows higher Δ*T*_max_ than those reported for P(dcbp-*co*-cpdt)/PEDOT [32], P(dcbp)/PEDOT [33], P(dcbp-*co*-bt)/PEDOT [34], P(bmco)/PEDOT [35], P(BCz-*co*-ProD)/tri-l poly(3,4-ethylenedioxythiophene)-poly(styrene sulfonic acid) (PEDOT-PSS) [31], and P(BCz-*co*-In)/PProDOT-Et_2_ ECDs [36]. 

Moreover, the comparison of η_max_ with reported ECDs revealed that P(tCz-co-DTC)/PProDOT-Me_2_ ECD showed higher η_max_ than those reported for P(dcbp-co-cpdt)/PEDOT [32] and P(dcbp-co-bt)/PEDOT ECDs [34]. However, P(tCz-co-DTC)/PProDOT-Me_2_ ECD showed lower η_max_ than those reported for P(BCz-co-ProD)/tri-l PEDOT-PSS [31] and P(BCz-co-In)/PProDOT-Et_2_ ECDs [36]. 

### 3.7. Open Circuit Memory

**Figure 9:**
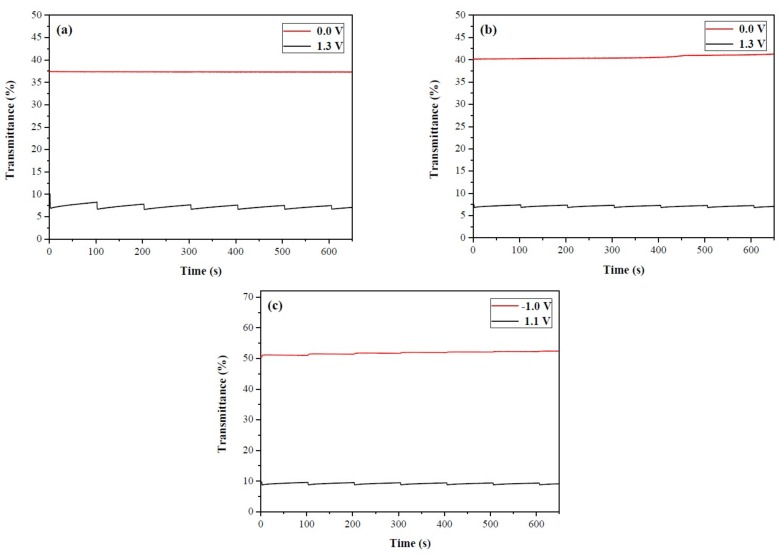
Open circuit stability of (**a**) PtCz/PProDOT-Me_2_; (**b**) P(tCz-*co*-bTP)/PProDOT-Me_2_; and (**c**) P(tCz-*co*-DTC)/PProDOT-Me_2_ ECDs.

The open circuit memory of ECDs is a crucial property due to it is related to the energy-saving of ECDs [37,38]. The open circuit stability of PtCz/PProDOT-Me_2_, P(tCz-*co*-bTP)/PProDOT-Me_2_, and P(tCz-*co*-DTC)/PProDOT-Me_2_ ECDs as shown in Figure 9a–c was detected at 583, 628, and 624 nm, respectively, by applying potentials at bleached and colored states for 1 s at each 100 s time interval. PtCz/PProDOT-Me_2_, P(tCz-*co*-bTP)/PProDOT-Me_2_, and P(tCz-*co*-DTC)/PProDOT-Me_2_ ECDs were nearly no transmittance change at bleached state. However, the open circuit stability of PtCz/PProDOT-Me_2_, P(tCz-*co*-bTP)/PProDOT-Me_2_, and P(tCz-*co*-DTC)/PProDOT-Me_2_ ECDs at colored state were less stable than that at bleached state. The transmittance changes of these ECDs were less than 5% at the colored state, indicating that PtCz/PProDOT-Me_2_, P(tCz-*co*-bTP)/PProDOT-Me_2_, and P(tCz-*co*-DTC)/PProDOT-Me_2_ ECDs would not need a refreshing current for retaining their colors in bleached and colored states.

### 3.8. Stability

**Figure 10:**
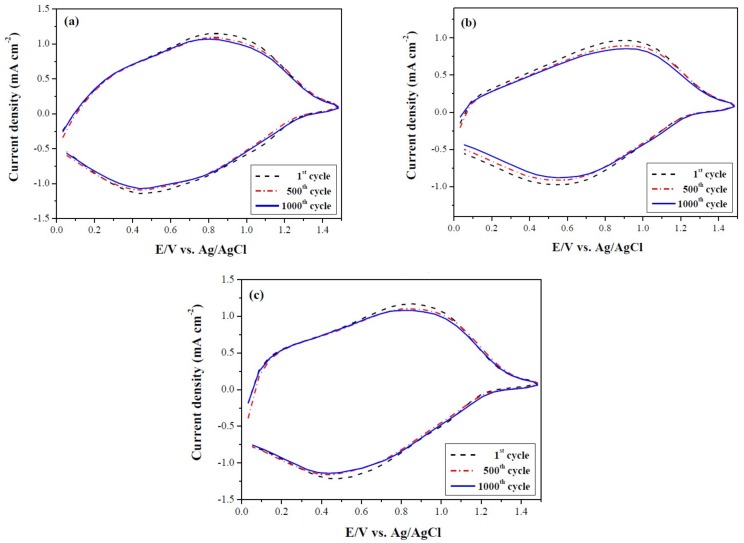
Cyclic voltammograms of (**a**) PtCz/PProDOT-Me_2_; (**b**) P(tCz-*co*-bTP)/PProDOT-Me_2_; and (**c**) P(tCz-*co*-DTC)/PProDOT-Me_2_ ECDs at the first, 500th, and 1000th cycles.

The redox stabilities of PtCz/PProDOT-Me_2_, P(tCz-*co*-bTP)/PProDOT-Me_2_, and P(tCz-*co*-DTC)/PProDOT-Me_2_ ECDs were monitored by CV at 1st, 500th and 1000th cycles [39,40], the scan rate was 500 mV s^−^^1^. As shown in Figure 10, 96.0%, 93.6%, and 96.7% of their electroactivities were maintained after the 500th cycle for PtCz/PProDOT-Me_2_, P(tCz-*co*-bTP)/PProDOT-Me_2_, and P(tCz-*co*-DTC)/PProDOT-Me_2_ ECDs, respectively, and 95.5%, 90.3%, and 96.0% of their electroactivities were maintained after the 1000th cycle for PtCz/PProDOT-Me_2_, P(tCz-*co*-bTP)/PProDOT-Me_2_, and P(tCz-*co*-DTC)/PProDOT-Me_2_ ECDs, respectively. Considering these results, ECDs employ P(tCz-*co*-bTP) and P(tCz-*co*-DTC) as anodic polymer films show potential for use in auto-dimming car mirror and motorcycle helmet-visors.

## 4. Conclusions

Copolymers (P(tCz-*co*-bTP), P(tCz-*co*-CPDT), P(tCz-*co*-DTC), and P(tCz-*co*-CPDTK)) were copolymerized electrochemically. Spectroelectrochemical investigations showed that P(tCz-*co*-CPDT) film revealed three different colors (light grey, green, and dark blue) at various voltages, whereas the color variations of P(tCz-*co*-DTC) film were light yellow at 0.0 V, yellow at 0.6 V, blue at 0.8 V, and black at 1.3 V. Five dual-type ECDs based on PtCz, P(tCz-*co*-bTP), P(tCz-*co*-CPDT), P(tCz-*co*-DTC), and P(tCz-*co*-CPDTK) films as anodic polymers and PProDOT-Me_2_ as the cathodic polymer were constructed and their electrochromic properties were characterized. P(tCz-*co*-bTP)/PProDOT-Me_2_ ECD showed high Δ*T*_max_ (32% at 628 nm) and fast switching time (less than 0.4 s), whereas P(tCz-*co*-DTC)/PProDOT-Me_2_ ECD revealed high Δ*T*_max_ (45.9% at 624 nm), high open circuit stability, and high redox stability after 1000 cycles, which makes P(tCz-*co*-bTP) and P(tCz-*co*-DTC) promising anodic copolymer films for ECDs’ applications.

## Figures and Tables

**Table 1:** Feed species of anodic polymer electrodes.

Anodic Polymer Electrodes	Feed Species of Anodic Polymers	Feed Molar Ratio of Anodic Polymers
PtCz	2 mM tCz	Neat tCz
P(tCz-*co*-bTP)	2 mM tCz + 2 mM bTP	tCz:bTP = 1:1
P(tCz-*co*-CPDT)	2 mM tCz + 2 mM CPDT	tCz:CPDT = 1:1
P(tCz-*co*-DTC)	2 mM tCz + 2 mM DTC	tCz:DTC = 1:1
P(tCz-*co*-CPDTK)	2 mM tCz + 2 mM CPDTK	tCz:CPDTK = 1:1

**Table 2:**
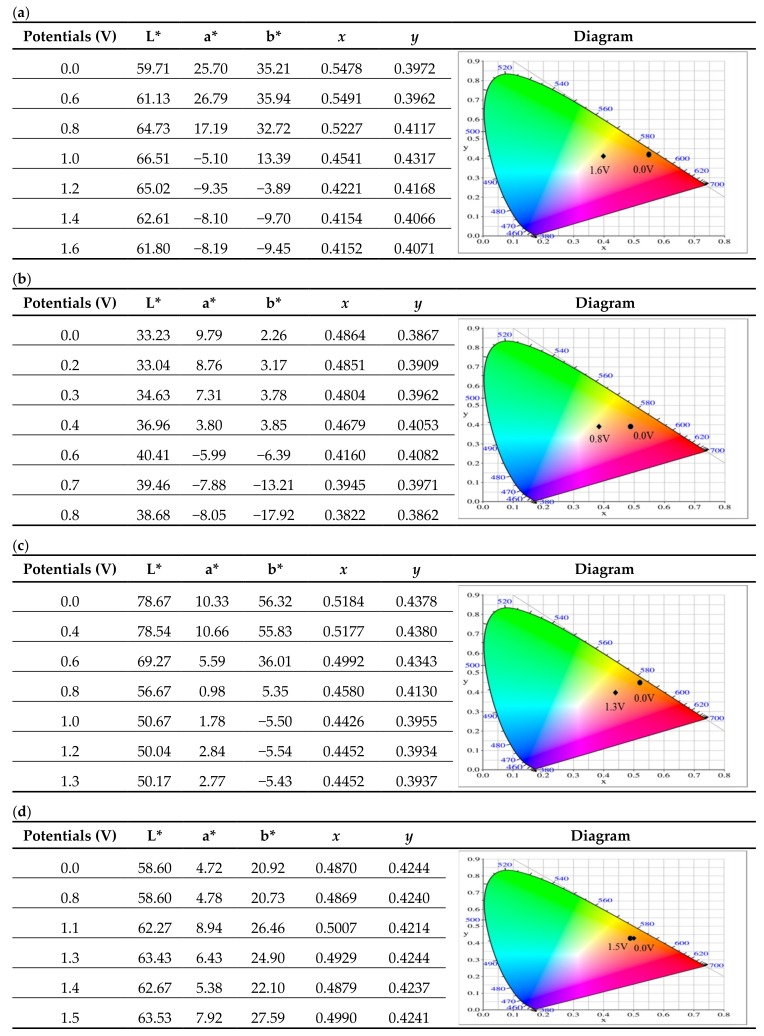
Colorimetric values of (**a**) P(tCz-*co*-bTP); (**b**) P(tCz-*co*-CPDT); (**c**) P(tCz-*co*-DTC); and (**d**) P(tCz-*co*-CPDTK) at various potentials.

**Table 3:** Electrochromic switching properties of the electrodes.

Electrodes	*λ* (nm)	*T*_ox_	*T*_red_	Δ*T*	△OD	*Q*_d_ (mC cm^−2^)	*η* (cm^2^ C^−1^)	*τ*_c_ (s)	*τ*_b_ (s)
PtCz	760	33.0	63.5	30.5	0.284	6.660	42.6	6.0	5.0
P(tCz-*co*-bTP)	967	9.5	52.5	43.0	0.742	10.986	67.6	6.5	5.0
P(tCz-*co*-CPDT)	864	13.5	42.2	28.7	0.494	6.453	76.7	6.5	5.5
P(tCz-*co*-DTC)	870	10.0	53.6	43.6	0.729	9.310	78.3	6.5	4.5
P(tCz-*co*-CPDTK)	984	19.5	44.0	24.5	0.353	5.653	62.5	5.5	3.5

**Table 4:** Transmittance changes and colouration efficiencies of carbazole-based polymer films.

Polymer Films	Δ*T*_max_ (%)	*η* (cm^2^ C^−1^)	Ref.
PETCB	36 (1100 nm)	-	[15]
PMCzP	29 (460 nm)	-	[30]
PBCz	18.6 (1050 nm)	180.3	[31]
P(NO_2_-3Cz)	52 (710 nm)	35	[14]
P(tCz-*co*-DTC)	43.6 (870 nm)	78.3	This work

**Table 5:**
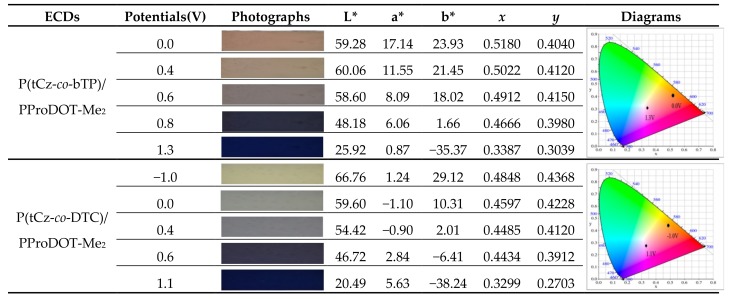
Photographs and colorimetric values of ECDs at different potentials.

**Table 6:** Electrochromic properties of the devices.

Devices	*λ* (nm)	*T*_ox_	*T*_red_	Δ*T*	ΔOD	*Q*_d_ (mC cm^−2^)	*η* (cm^2^ C^−1^)	*τ*_c_ (s)	*τ*_b_ (s)
PtCz/PProDOT-Me_2_	583	6.6	37.5	30.9	0.754	1.724	437.4	0.9	0.4
P(tCz-*co*-bTP) /PProDOT-Me_2_	628	6.7	38.7	32.0	0.761	1.968	387.0	0.4	0.2
P(tCz-*co*-CPDT) /PProDOT-Me_2_	582	7.4	27.1	19.7	0.563	1.343	419.7	0.3	0.5
P(tCz-*co*-DTC) /PProDOT-Me_2_	624	8.6	54.5	45.9	0.801	2.001	400.5	1.2	1.6
P(tCz-*co*-CPDTK) /PProDOT-Me_2_	582	7.6	37.1	29.5	0.688	1.342	513.0	0.6	0.2

**Table 7:** Δ*T* and *η* of ECDs.

ECD Configurations	Δ*T*_max_ (%)	*η*_max_ (cm^2^ C^−1^)	Ref.
P(dcbp-*co*-cpdt)/PEDOT	39.8 (628 nm)	319.98 (628 nm)	[32]
P(dcbp)/PEDOT	19 (550 nm)	-	[33]
P(dcbp-*co*-bt)/PEDOT	28.6 (700 nm)	234 (700 nm)	[34]
P(bmco)/PEDOT	35 (620 nm)	-	[35]
P(BCz-*co*-ProD)/tri-l PEDOT-PSS	41 (642 nm)	417 (642 nm)	[31]
P(BCz-*co*-In)/PProDOT-Et_2_	42.0 (587 nm)	634 (587 nm)	[36]
P(tCz-*co*-DTC)/PProDOT-Me_2_	45.9 (624 nm)	401 (624 nm)	This work
